# Cortisol Secretion and Subsequent Impaired Lymphopoiesis after Starvation Can Be Reduced by Moxibustion Treatment

**DOI:** 10.1155/2021/8856687

**Published:** 2021-02-03

**Authors:** Kyung Ho Hwang, Kiyoung Jang, Sang-Yun Nam, Yong Ju Kim

**Affiliations:** ^1^Department of Oriental Medicine Resources, Jeonbuk National University, Iksan 54596, Republic of Korea; ^2^Department of Lifestyle Medicine, Jeonbuk National University, Iksan 54596, Republic of Korea; ^3^Department of Environmental Science and Biotechnology, Jeonju University, Jeonju 55069, Republic of Korea

## Abstract

As a known steroid hormone, cortisol is involved in gluconeogenesis. Uninterrupted cortisol secretion has fatal effects, both physically and psychologically, because cortisol counteracts the immune response. Moxibustion (Mox) treatment is a traditional technique used in East Asia, which therapeutically transfers heat to certain points on the body surface. In the present study, the effect of Mox treatment on stress hormone secretion was investigated using a mouse model of starvation, in which Mox was applied on the Zhongwan acupoint (CV12). First, high cortisol levels induced by starvation were dose-dependently reduced by Mox treatment. In addition, the stress-induced decline in lymphoid progenitor cell production accompanied by altered cellularity in the thymus, bone marrow, and spleen was also significantly recovered by Mox treatment. Taken together, these findings indicated that Mox treatment reduces stress hormone secretion, which may rescue stress-induced lymphopoiesis impairment. These observations also suggested that enhanced resistance to stress may be one of the mechanisms underlying the immunomodulatory effects of Mox treatment.

## 1. Introduction

Cortisol is generally involved in gluconeogenesis, which stimulates glucose synthesis from noncarbohydrate sources [1]. It functions to increase glucose concentration and also prevents tissues from taking the glucose from the blood by decreasing the sensitivity of peripheral tissue to insulin [2]. When cortisol is released in excessive amounts in the short or long term, it can have a fatal effect both physically and psychologically [3]. Cortisol has been inversely correlated with lymphopoiesis because it prevents T and B lymphocyte proliferation [4], suggesting that cortisol may inhibit the immune response. Because cortisol is also known as a stress response hormone, controlling it with alternative therapies rather than pharmacotherapy may be more useful [5–7].

Moxibustion (Mox) is a traditional therapeutic technique that has been used in many regions of East Asia, including China, Korea, and Japan, for over 2,000 years. The World Health Organization defines Mox as a therapeutic procedure involving the application of ignited material to transfer heat to certain points of the body surface for treating diseases by regulating the function of meridians or channels and visceral organs [8, 9]. Mox can be used alone or in combination with acupuncture as an alternative modality for treating cancer, infectious diseases, rheumatoid arthritis, ulcerative colitis, asthma, hypertension, and pain [10]. Although Mox treatment is considered as an alternative treatment that has been used for a long time in the Oriental Medicine, drawing conclusions about its immunomodulatory effects is difficult because results of studies on the effects of Mox treatment are limited and inconsistent [11, 12]. Additionally, greater understanding about the optimal clinical application of Mox, including the size of Mox cones and the duration or number of treatment and applied acupuncture points, remains to be elucidated. Recent systematic reviews of randomized controlled trials support the notion that Mox has potential benefits in treating several diseases [13–15].

In the present study, we report one of the effects of Mox treatment using a starvation mouse model. The serum cortisol level of starved mice was higher than that of normal mice. In agreement with this, the numbers and characteristics of thymic, bone marrow, and splenic cells were decreased by starvation. However, Mox treatment rescued their cortisol levels, as well as lymphopoiesis. It is the first time that Mox treatment has been reported to reduce stress hormone secretion, which may have potential applications in rescuing stress-induced impairment of lymphopoiesis.

## 2. Materials and Methods

### 2.1. Animals

Male Balb/*c* mice (23 ± 2 g) were provided by the Orient Co., (Seoul, Korea) and housed under constant conditions (temperature, 25°C ± 1°C; humidity, 45% ± 5%; light/dark cycle, 12/12 h). The animals had free access to food pellets and sterile tap water. The protocol for this study was approved by Institutional Animal Care and Use Committee of Jeonju University (approval number: JJU-IACUC-2015-02). All procedures were performed in accordance with the guidelines according to the National Institute for Health's “Guide for the Care and Use of Laboratory Animals” (National Academies Press, Washington DC, USA).

### 2.2. Starvation and Experimental Design

**Figure 1:**
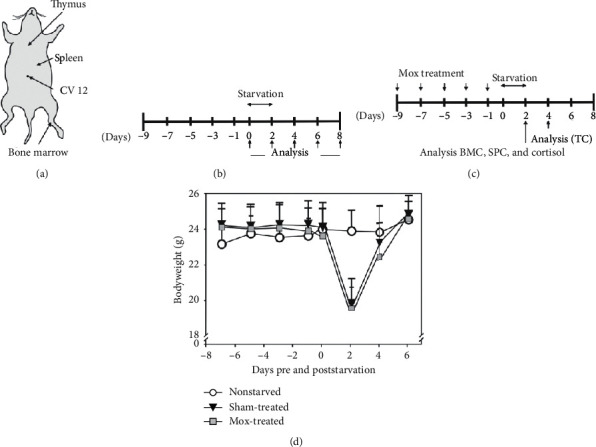
Experimental design. (a) The representation of Mox treatment (CV12) and lymphoid organ (thymus, spleen, and bone marrow). (b) The time course of changes in cell number and cellularity of lymphoid organs during starvation; a nonstarved group was sacrificed on day 0, whereas four starved groups were sacrificed at 2, 4, 6, or 8 d following starvation, and lymphopoiesis was subsequently analyzed. (c) The effects of Mox treatment; five Mox-treated groups were treated 1, 2, 3, 4, or 5 times. These five groups and the sham-treated groups were then starved 1 d following the final treatment. Lymphopoiesis was analyzed on day 2 or 4, when the decline in cell number was most prominent. (d) Changes in bodyweight during and following Mox treatment and starvation (*n* = 8). The nonstarved mice group is marked with empty circles, whereas the starved mice group is marked with black triangles (sham-treated) and gray quadrangles (Mox-treated).

For simulating starvation, 1 d following the final Mox treatment, sham-treated controls and Mox-treated mice were placed in clean cages without food for 48 h; water was provided ad libitum. First, to examine the time course of changes in cell number and cellularity of lymphoid organs during starvation, 40 mice were randomly divided into five different groups. The mice in one nonstarved group were sacrificed on day 0, whereas four starved groups were sacrificed at 2, 4, 6, or 8 d following starvation, and lymphopoiesis was subsequently analyzed (*n* = 8 mice/group; Figure 1(b)).

Second, to examine the effects of Mox treatment, 56 mice were randomly divided into the following seven groups: (a) nonstarved, (b) starved and sham-treated, and (c) five starved and Mox-treated groups, wherein mice underwent Mox treatment 1, 2, 3, 4, or 5 times (*n* = 8 mice/group). At 1 d, following the final treatment, mice were starved for 2 d more. Two independent sets of experiments were performed, and the results were analyzed on days 2 (bone marrow and spleen) and 4 (thymus and serum cortisol), since it is at these timepoints where the decline in cell number or elevation of the serum level following starvation was most prominent (Figure 1(c)). Individual bodyweights were measured using an Adventurer Pro electronic scale (Ohaus Corporation, Pine Brook, NJ, USA) and recorded at intervals of 1-2 d to monitor animal health and response to starvation and Mox treatment.

### 2.3. Mox Treatment

Thermal stimulation using Mox cones (direct Mox) was applied to a point anatomically equivalent to the CV12 acupuncture point (Zhongwan) in humans. This is located along the anterior median line of the upper abdomen, 10 mm below the xiphisternal synchondrosis (Figure 1(a)), based on the Atlas of Animal Points (Institute of Experimental Acupuncture Research of China) [16].

For anesthesia, 2,2,2-tribromoethanol (Aldrich Chemical, Milwaukee, WI, USA) dissolved in tert-amyl alcohol (Sigma–Aldrich, St Louis, MO, USA) was used at a dose concentration of 50 mg/kg bodyweight. Mice were anesthetized through intraperitoneal injection, and the hair around the acupuncture points (2 × 2 cm) was shaved to expose the local skin. Half-rice-sized (0.5 ± 0.025 mg) (Figure S1A) Mox cone particles were placed directly on the shaved skin surface at the acupuncture point and were ignited (Figure S1B and C). After placing the Mox cone on the CV12, burning it, it took about 5 seconds to burn completely (Figure S1D). The ashes of the previously burned Mox cone were left as they were, and the new Mox cone was placed in the CV12 position and lit again. All of these processes were applied 5 times. Mox treatment was repeated every other day up to five times, and sham controls were handled in the same manner except that the cones were not ignited. To standardize the conditions, the mice were treated with Mox between 9 : 00 and 10 : 00 a.m.

### 2.4. Measurement of Serum Cortisol Levels Enzyme-Linked Immunosorbent Assay (ELISA)

Serum samples were first collected from blood samples following centrifugation for 10 min (2000 x g and 4°C) and stored at −20°C until cortisol level analysis. ELISAs for cortisol level estimation were performed using the cortisol-ELISA kit (B-Bridge International, San Jose, CA, USA), according to the manufacturer's instructions.

### 2.5. Collection of Cells and Cell Counts

Anesthetized mice were first sacrificed through cardiac puncture before the thymus and spleen were removed. Single-cell suspensions were prepared by forcing organs with curved needles. BMCs were harvested from both femurs by flushing with 5 mM ethylenediaminetetraacetic acid-phosphate buffered saline (PBS, pH 7.4) containing 0.5% bovine serum albumin. Following centrifugation for 10 min (400 × g and 4°C), viable TC and BMC counts were determined through trypan blue exclusion staining using a hemocytometer. SPC counts were obtained following appropriate dilution with Turk's solution (0.05% gentian violet plus 2% acetic acid).

### 2.6. Immunofluorescence Staining and Flow Cytometric Analysis

The harvested cells were incubated with 10% rat anti-mouse CD16/32 (Fc block, clone 93; eBioscience, San Diego, CA, USA) in PBS containing 0.5% bovine serum albumin for 30 min and then stained with surface marker-specific monoclonal antibodies purchased from eBioscience. The following antibodies were applied: anti-CD3-fluorescein isothiocyanate (FITC) (clone 145-2C11), anti-CD4-FITC (clone GK1.5), anti-CD8-phycoerythrin (PE) (clone 53.6.7), anti-CD19-PE (clone 1D3), anti-CD19-PerCP-Cy5.5 (clone 1D3), anti-mouse IgM-PE (clone II/41), and anti-mouse IgD-FITC (clone 11–26c). Flow cytometry data were obtained using a fluorescence-activated flow cytometer (FACSort, Becton, Dickinson and Co., Mountain View, CA, USA) with the BD CellQuest software (BD Biosciences, Becton Dickinson and Company). Data were analyzed using the WinMDI software (Scripps Institute, La Jolla, CA, USA).

### 2.7. Statistical Analysis

Statistical analysis was performed using the SPSS Statistics for Windows software (version 23.0; SPSS Inc., Chicago, IL, USA). Data were presented as the means ± standard error of the mean and were analyzed using one-way analysis of variance (ANOVA). Significant results obtained following ANOVA were further analyzed for significance using Fisher's least significant difference multiple-comparison post hoc test. *P* values <0.05 were considered as significantly different.

## 3. Results

### 3.1. Effects of Mox Treatment on Cortisol Secretion in Starved Mice

Cortisol is secreted under stress conditions, such as starvation. Therefore, we separated the nonstarved group from the starved group. The bodyweight of the nonstarved group slightly increased from 23.2 ± 0.9 g to 23.9 ± 1.1 g. Starvation for 2 d resulted in a substantial weight loss of 17%–18% (sham-treated, from 24.2 ± 1.3 to 19.8 ± 1.5 g; Mox-treated, from 24.1 ± 1.3 to 19.6 ± 1.2 g); however, there was no significant difference. The maximum weight loss was also similar in both sham-treated (6.0 g; from 26.0 to 20.0 g) and Mox-treated (6.2 g; from 25.2 to 19.0 g) groups. Additionally, almost full recovery was similarly observed following 4 d of refeeding (day 6; Figure 1(d)).

**Figure 2:**
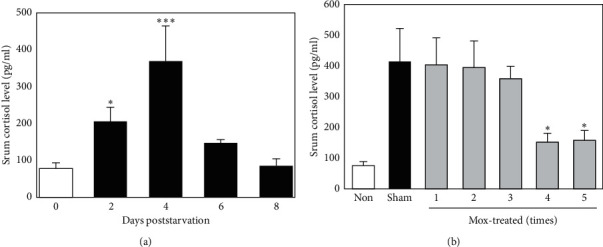
Effects of Mox treatment on serum cortisol levels. (a) Time course of changes in serum cortisol levels following starvation. Mice were starved for 2 (d) and serum cortisol levels were measured every other day. *∗P* < 0.05; *∗∗∗P* < 0.001 versus the baseline level on day 0 (*n* = 8). (b) Effects of Mox treatment on serum cortisol levels. Mice were sham- or Mox-treated five times over 2-d intervals (days −9, −7, −5, −3, and −1) and then starved from day 0 to 2. On day 4, serum cortisol levels were examined using ELISA. *∗P* < 0.05 versus sham-treated controls (*n* = 8).

To confirm whether starvation affects cortisol levels, we investigated serum cortisol levels following starvation. Significantly higher levels of serum cortisol were observed at 2 d following starvation in the starved group, compared with that of the nonstarved group. Cortisol level peaked on day 4 and then rapidly decreased, whereas higher levels were maintained until day 6 (Figure 2(a)). To confirm the effects of Mox treatment on cortisol levels, mice were treated up to five times with 2-d intervals (days −9, −7, −5, −3, and −1) before starvation. Interestingly, Mox treatment significantly reduced serum cortisol levels in a dose-dependent manner, and the levels in mice receiving four treatments were significantly lower than those of the sham-treated controls (Figure 2(b)). As five treatments exerted no additional beneficial effect, Mox treatment was performed four times in all subsequent experiments.

### 3.2. Effects of Mox Treatment on Primary Lymphatic Cell Number and Differential Counts

**Figure 3:**
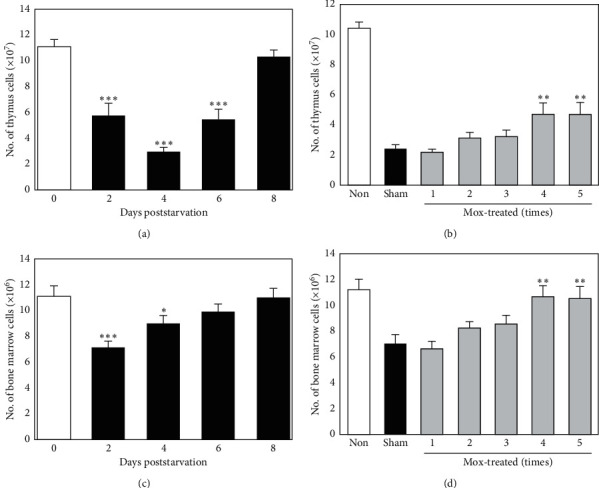
Effects of Mox treatment on the number of TCs and BMCs. (a) Time course of changes in TC count following starvation. Mice were starved for 2 (d), and TCs were counted every other day. *∗∗∗P* < 0.001 versus nonstarved controls (*n* = 8). (b) Dose response of Mox treatment on TC number restoration. Mice were sham- or Mox-treated five times over 2-d intervals (days −9, −7, −5, −3, and −1) and then starved from day 0 to 2. On day 4, TC cells were counted. *∗∗P* < 0.01 versus sham-treated controls (*n* = 8). (c) Time course of changes in BMC count following starvation. Mice were starved for 2 (d), and BMCs were counted every other day. *∗P* < 0.05; *∗∗∗P* < 0.001 versus nonstarved controls (*n* = 8). (d) Dose response of Mox treatment on the BMC number restoration. Mice were sham- or Mox-treated five times over 2-d intervals (days −9, −7, −5, −3, and −1) and then starved from day 0 to 2 (d). On day 2, BMCs were counted. *∗∗P* < 0.01 versus sham-treated controls (*n* = 8).

It is well known that lymphopoiesis is impaired by cortisol. Therefore, we hypothesized that Mox treatment may subsequently rescue stress-induced lymphopoietic impairment. Thymus cells (TCs) are well-established to be committed to apoptosis following exposure to stress hormones. Therefore, TC count changes following starvation were examined. As expected, in the starved group, starvation resulted in a rapid decline in TC count on day 2, compared with nonstarved mice, and the sharp decline continued even after refeeding until 4 d following starvation (Figure 3(a)). Thereafter, the TC count in starved mice gradually recovered and were completely restored by day 8. Mox treatment alleviated the decline in TC numbers in a dose-dependent manner, and the TC count in mice receiving four treatments was significantly higher compared with the sham-treated controls (Figure 3(b)).

In the starved group, starvation also reduced bone marrow cell (BMC) count on day 2, compared with nonstarved mice (Figure 3(c)). BMC count was gradually restored; however, the level remained lower than the control group on day 4. The BMC count at 6 days following starvation was comparable with that of the nonstarved mice group. Mox treatment prevented the decline in BMC count in a dose-dependent manner. Additionally, the number of BMCs in mice receiving four or five Mox treatments was significantly higher than the sham-treated controls, and the mice in these groups demonstrated full recovery (Figure 3(d)).

### 3.3. Flow Cytometry Assay of TCs and BMCs

To investigate the effects of Mox treatment on TCs and BMCs in more detail, the cellularity of TCs was assessed on day 4 through flow cytometry. As indicated in Table 1, starvation resulted in a substantial decline in double-positive (DP) and CD8^+^ cell subpopulations but not double-negative and CD4^+^ cell subpopulations. The number of DP cells in the sham-treated controls (0.85 ± 0.16 × 10^7^ cells) was notably equivalent to 9.1% of the number in nonstarved mice (9.18 ± 0.48 × 10^7^ cells). On average, the proportion of DP cells in the TC population decreased from 79.5% to 31.4% following starvation (Figure S2A and B). Mox treatment significantly alleviated the decline in DP cell count, compared with sham-treated controls, and their relative proportions were also restored to 62.5% following Mox treatment (Figure S2C). The number of single CD8^+^ cells significantly declined following starvation; however, this was not altered by Mox treatment (Table 1), despite the increase in proportion (Figure S2A and B).

BMCs were assessed 2 d following starvation through flow cytometry, which included the detection of B lineage (CD19^+^), pre/pro (IgM^−^ IgD^−^), immature (IgM^+^ IgD^−^), and mature (IgM^+^ IgD^+^) B cell subpopulations. As shown in Table 2, starvation resulted in a significant decline in CD19^+^ B lineage numbers (3.48–1.37 × 10^6^ cells) and their proportions in whole BMC counts (30.8%–19.1%; Figure S3A and B), suggesting that Mox treatment markedly alleviated the decline to 2.44 × 10^6^ cells and 22.5% (Figure S3C), respectively.

All B subpopulations, apart from mature B cells (IgM^+^ IgD^+^), were also reduced in response to starvation (Table 2 and Figure S3D). Notably, the number of pre/pro (IgM^−^ IgD^−^) B cells was significantly decreased in starved mice compared with nonstarved mice (0.4 × 10^7^ vs. 2.19 × 10^7^ cells, respectively). Table 2 indicates that this decline was significantly inhibited by Mox treatment (1.26 × 10^7^ cells). Immature B cell (IgM^+^ IgD^−^) numbers were also significantly reduced following starvation (0.87–0.34 × 10^7^ cells) but were significantly restored by Mox treatment; however, mature cell numbers were unaltered following starvation for 2 d, and the relative proportion in BMC counts was increased from 3.6% to 7.9% (Figure S3D and E) and partially restored by Mox treatment (Figure S3F).

### 3.4. Effects of Mox Treatment on Secondary Lymphatic Cell Number and Differential Counts

**Figure 4:**
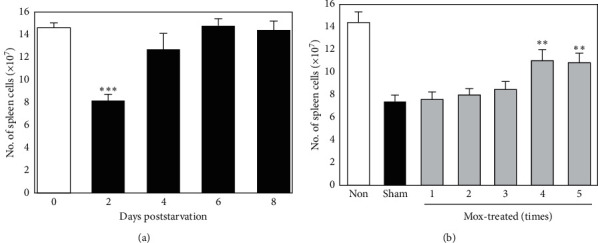
Effects of Mox treatment on SPC count. (a) Time course of changes in SPC count following starvation. Mice were starved for 2 (d), and SPCs were counted every other day. *∗∗∗P* < 0.001 versus nonstarved controls (*n* = 8). (b) Dose response of Mox treatment on SPC number restoration. Mice were sham- or Mox-treated five times over 2-day intervals (days −9, −7, −5, −3, and −1) and then starved from day 0 to 2. On day 2, SPCs were counted. *∗∗P* < 0.01 versus sham-treated controls (*n* = 8).

Based on the results, we confirmed that Mox treatment may partially rescue impaired lymphopoiesis. We investigated the change in cell number and cellularity of spleen cells (SPCs, a secondary lymphoid tissue). In starved mice, starvation for 2 d resulted in a significant decrease in SPC number, compared with nonstarved mice, but was rapidly recovered (Figure 4(a)).

To determine the dose response of Mox treatment, SPCs were analyzed 2 d following starvation, when the smallest number of SPCs was observed. Mox treatment prevented the decline in the SPC count in a dose-dependent manner (Figure 4(b)), and the SPC numbers in mice receiving four treatments were significantly higher than those of the sham-treated controls. For a more detailed evaluation of the results, T and B cell distribution in the total SPC count was assessed 2 d following starvation. B cells were dominant in the spleen of nonstarved mice, with an average T/B cell ratio of 0.77 (Table 3). Starvation resulted in a substantial decline in T and B cells; however, the decline was more prominent in B cells than in T cells, reversing the T/B ratio to 1.31. The proportion of B cells in the total SPC count decreased from 48.6% to 39.9% on average (Figure S4A and B) at 2 d following starvation, which was lower than that of T cells (51.9%). However, the decline in the number of B cells was significantly reduced by Mox treatment, compared with the sham-treated controls, and the T/B cell ratio (0.73) was also restored to a level comparable with that of nonstarved mice. The proportion of B cells was also restored by Mox treatment (51.9% of the total SPC count; Figure S4C).

## 4. Discussion

Although recent systematic reviews of randomized controlled trials have indicated that Mox treatment may help control several diseases [13, 17–19], the mechanisms underlying Mox treatment's effects remain to be elucidated. In addition, there is limited information regarding the adverse effects and safety of Mox treatment, which is a long-standing issue that needs to be resolved [20]. Identifying its mechanism of action may provide an important foundation for the extended use of Mox. Regarding the effects of Mox on immune response, previous studies suggested that Mox inhibits inflammation in vitro [14] and in vivo [15]. A reduction in the expression of tumor necrosis factor-*α* and p38 mitogen-activated protein kinase was proposed as the potential mechanism [15]. Several data sources demonstrated that Mox enhances T and NK cell activities [21–23]. Recent microarray analysis of skin tissue obtained from the Mox site indicated that Mox exerts substantial immunomodulating abilities, and the signaling pathways involved are very complex [24]. Studies have identified differences between the signaling pathways activated in response to Mox treatment under normal physiologic and inflammatory conditions, the latter of which was induced by Freund's complete adjuvant injection. Differentially expressed genes in an inflammatory condition were observed to be part of the signaling pathways and biological processes involved in immunity, whereas those under normal physiologic conditions were involved in metabolism [24].

It was hypothesized that Mox might modulate immunity via a neuroimmune circuit, and Mox treatment's effects on lymphopoiesis were studied. To prove this hypothesis, a mice starvation model was applied, and Mox was treated at CV12. The acupuncture point CV12 is a front-mu point of the stomach, which is exterior-interiorly associated with the spleen. The stomach and spleen produce a vital energy source (qi) and blood in traditional Chinese medicine [25]. As such, CV12 has been used to treat cancer-associated fatigue [26] or improve immune response in cancer patients [27]. CV12 was therefore selected in the present study. Mox was applied to the region directly, which caused a mild inflammation on the skin. Whether this inflammatory response enhanced lymphopoiesis may be questioned; however, it should be noted that inflammation has been demonstrated to regulate the normal balance of granulopoiesis and lymphopoiesis by increasing lymphopoiesis suppression [28].

Starvation induces the elevation of serum cortisol levels and decreases lymphopoiesis. Starvation induces the elevation of serum cortisol levels and decreases lymphopoiesis. These mechanisms are similarly observed in the other stress-induced models reported previously [29–31]. Starvation stress resulted in a more than fourfold increase in cortisol secretion by day 4 of starvation. The most severe lymphopoietic impairment was observed at 2 days in the bone marrow and 4 days in the thymus after starvation. At these time points, the reduced immature cell numbers were more prominent. This is consistent with previous results demonstrating that early B cells [32] and DP thymocytes are more sensitive to glucocorticoids [33]. Interestingly, several studies reported that Mox treatment controls the cortisol levels. Given that cortisol acts as a mediator in various mechanisms in the body, some diseases are associated with abnormally low levels of such hormone. In the present study, Mox treatment normalized low cortisol levels. Therefore, Mox treatment can potentially regulate abnormal cortisol secretion [34, 35].

This research did not ascertain whether reduced cortisol levels directly improve lymphopoiesis in starved mice. However, it is well understood that stress hormones such as glucocorticoids impair lymphopoiesis, which can be observed during starvation [36]. Therefore, Mox treatment reduces stress hormone secretion and may subsequently rescue stress-induced lymphopoiesis impairment. This may be one of the mechanisms underlying Mox treatment's immunomodulatory effects, consistent with a previous study showing that Mox increases the thermal pain threshold and the minimum amount of stimulations that cause pain [37]. Thus, Mox exerts an analgesic effect and reduces stress hormone secretion [37]. This research is also supported by a recent study demonstrating that Mox displays an analgesic effect through the downregulation of transient receptor potential vanilloid type 1 and heat shock protein 70 expression in BMCs [38].

## 5. Conclusion

These results clearly demonstrated that Mox suppresses stress hormone secretion, thereby possibly rescuing stress-induced lymphopoiesis impairment. These observations also supported the notion that enhanced resistance to stress may be one of the mechanisms underlying the immunomodulatory effects of Mox treatment.

## Figures and Tables

**Table 1:** Effect of Mox treatment on the cellularity of thymus cells^††^.

Groups	Total count (×10^7^ cells)	Differential cell counts (×10^7^ cells)			
		Double negative	Double positive	CD4+	CD8+
Nonstarved	11.6 ± 0.68	0.51 ± 0.02	9.18 ± 0.48	1.29 ± 0.19	0.61 ± 0.08
Starved, sham-treated	2.58 ± 0.32^###^	0.41 ± 0.11	0.85 ± 0.16^###^	1.02 ± 0.10	0.31 ± 0.03^##^
Starved, Mox-treated	4.25 ± 0.56*∗*	0.34 ± 0.07	2.70 ± 0.42*∗∗*	0.88 ± 0.08	0.35 ± 0.02

^††^Mox treatment was performed four times, and then, mice were starved for 2 days. At 4 days, following the start of starvation, total cell counts of collected thymus cells were obtained. Differential cell counts of CD4^+^ and CD8^+^ cells were determined by flow cytometric analysis. Values are presented as the means ± standard error of the mean (*n* = 6). ##*P* < 0.01 and ###*P* < 0.001 vs. the nonstarved group. *∗P* < 0.05 and *∗∗P* < 0.01 vs. sham-treated controls. Mox, moxibustion.

**Table 2:** Effect of Mox treatment on the cellularity of bone marrow cells^††^.

Groups	Total counts (×10^6^ cells)	Differential cell counts (×10^6^ cells)			
		B lineage	Pre/Pro	Immature	Mature
Nonstarved	11.3 ± 0.99	3.48 ± 0.34	2.19 ± 0.31	0.87 ± 0.05	0.41 ± 0.05
Starved, sham-treated	7.00 ± 0.51^##^	1.37 ± 0.15^###^	0.47 ± 0.07^###^	0.34 ± 0.04^###^	0.56 ± 0.09
Starved, Mox-treated	10.9 ± 1.03*∗∗*	2.44 ± 0.23*∗*	1.26 ± 0.15*∗*	0.57 ± 0.10*∗*	0.61 ± 0.11

^††^Mox treatment was performed four times, and at 2 days, following the start of starvation, total cell counts of collected bone marrow cells were ascertained. Differential cell counts were determined using flow cytometric analysis, and the results for B lineage (CD19^+^), immature B (IgM^+^), and mature B (IgM^+^ IgD^+^) cells were collected. The number of pre/pro (IgM^−^ IgD^−^) cells was estimated by subtracting immature and mature B cell values from B lineage data. Values are presented as the means ± standard error of the mean (*n* = 5). ##*P* < 0.01 and ###*P* < 0.001 vs. the nonstarved group. *∗P* < 0.05 and *∗∗P* < 0.01 vs. sham-treated controls. Mox, moxibustion.

**Table 3:** Effect of Mox treatment on the cellularity of spleen cells^††^.

Groups	Total count (×10^7^ cells)	Differential cell counts (×10^7^ cells)		
		T cells	B cells	T/B cell ratio
Nonstarved	14.2 ± 1.39	5.32 ± 0.66	7.01 ± 0.97	0.77 ± 0.05
Starved, sham-treated	7.17 ± 0.76^###^	3.68 ± 0.32^#^	2.86 ± 0.31^###^	1.31 ± 0.06^###^
Starved, Mox-treated	9.53 ± 0.73*∗*	3.60 ± 0.32	4.94 ± 0.39*∗*	0.73 ± 0.03*∗∗∗*

^††^Mox treatment was performed four times, and at 2 days, following the start of starvation, the total cell counts of collected spleen cells were ascertained. Differential cell counts were determined by flow cytometric analysis, and the number of CD3^+^ T and CD19^+^ B cells was estimated. Values are presented as the means ± standard error of the mean (*n* = 6). #*P* < 0.05 and ###*P* < 0.001 vs. the nonstarved group. *∗P* < 0.05 and *∗∗∗P* < 0.001 vs. sham-treated controls. Mox, moxibustion.

## Data Availability

The datasets used/or analyzed during the current study are available from the corresponding author upon request.

## Abbreviations

Mox: Moxibustion
TC: Thymus cell
BMC: Bone marrow cell
SPC: Spleen cell.

## Supplementary Materials

Supplementary Materials Supplementary Figure 1. Representative flow cytometry plots showing the proportions (%) of TC subpopulations on day 4 (*n* = 6). Cells were analyzed with a two-color antibody combination of CD4 and CD8 for (A) nonstarved, (B) sham control, and (C) Mox-treated mice. Supplementary Figure 2. Representative flow cytometry plots showing the proportions (%) of BMC subpopulations on day 2 (*n* = 5). Cells were analyzed using a three-color antibody combination for CD19, IgD, and IgM for (A and D) nonstarved, (B and E) sham control, and (C and F) Mox-treated mice. Whole BMC counts were analyzed for (A–C) CD19+ cells, which were gated, and further analyzed for (D–F) IgD+/IgM+ cells. Supplementary Figure 3. Representative flow cytometry plots showing the proportions (%) of SPC subpopulations on day 2 (*n* = 6). Cells were analyzed using a two-color antibody combination of CD3 and CD19 for (A) nonstarved, (B) sham control, and (C) Mox-treated mice.
