# Supplementary material for: Cortisol Secretion and Subsequent Impaired Lymphopoiesis after Starvation Can Be Reduced by Moxibustion Treatment
Source: Evid Based Complement Alternat Med. 2021 Feb 3;2021:8856687. doi: 10.1155/2021/8856687 (PMC7878081; doi:10.1155/2021/8856687)
Supplement: Supplementary Materials — Supplementary Figure 1. Representative flow cytometry plots showing the proportions (%) of TC subpopulations on day 4 (n = 6). Cells were analyzed with a two-color antibody combination of CD4 and CD8 for (A) nonstarved, (B) sham control, and (C) Mox-treated mice. Supplementary Figure 2. Representative flow cytometry plots showing the proportions (%) of BMC subpopulations on day 2 (n = 5). Cells were analyzed using a three-color antibody combination for CD19, IgD, and IgM for (A and D) nonstarved, (B and E) sham control, and (C and F) Mox-treated mice. Whole BMC counts were analyzed for (A–C) CD19+ cells, which were gated, and further analyzed for (D–F) IgD+/IgM+ cells. Supplementary Figure 3. Representative flow cytometry plots showing the proportions (%) of SPC subpopulations on day 2 (n = 6). Cells were analyzed using a two-color antibody combination of CD3 and CD19 for (A) nonstarved, (B) sham control, and (C) Mox-treated mice. [file 8856687.f1.docx]

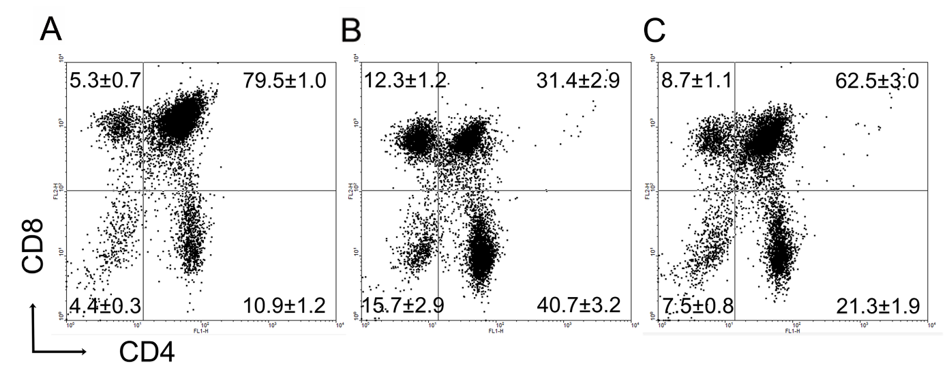


Supplementary Figure 1

Representative flow cytometry plots showing the proportions (%) of TC subpopulations on day 4 (n=6). Cells were analyzed with a two-color antibody combination of CD4, and CD8 for (A) non-starved, (B) sham control and (C) Mox-treated mice.


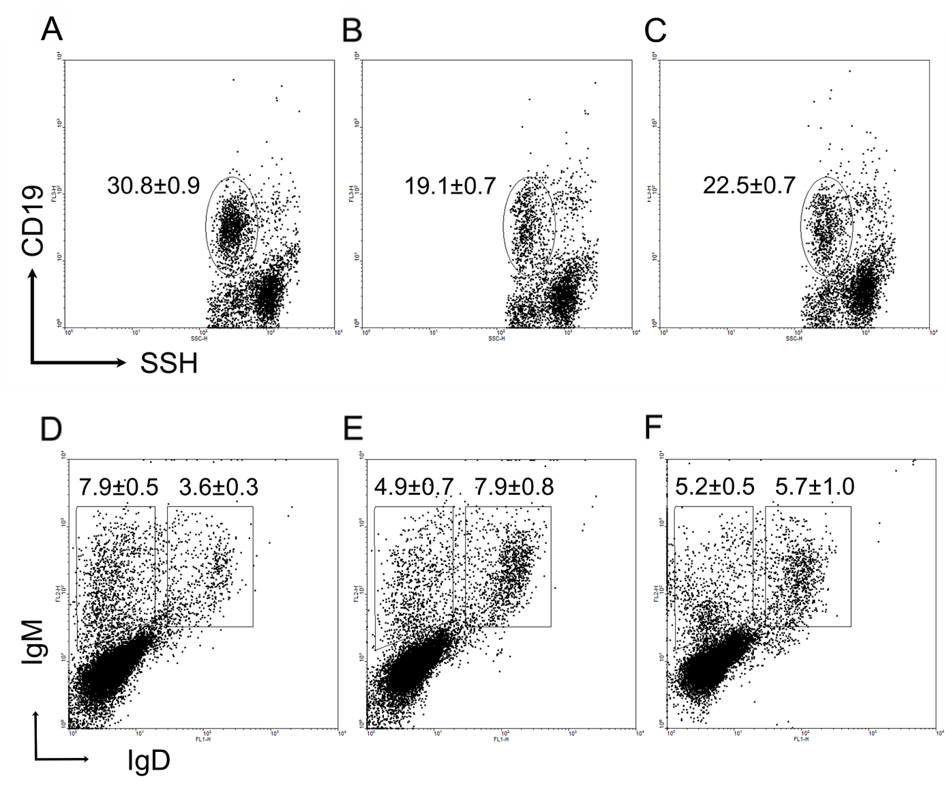


Supplementary Figure 2

Representative flow cytometry plots showing the proportions (%) of BMC subpopulations on day 2 (n=5). Cells were analyzed using a three-color antibody combination for CD19, IgD and IgM for (A and D) non-starved, (B and E) sham control and (C and F) Mox-treated mice. Whole BMC counts were analyzed for (A-C) CD19^+^ cells, which were gated and further analyzed for (D-F) IgD^+^/IgM^+^ cells.


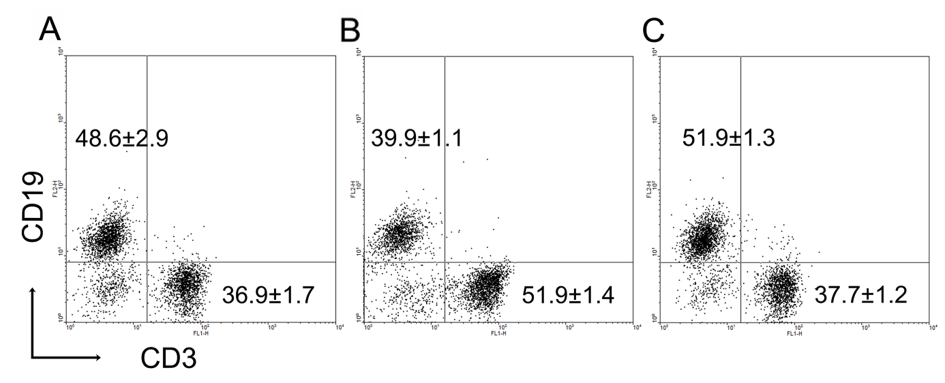


Supplementary Figure 3

Representative flow cytometry plots showing the proportions (%) of SPC subpopulations on day 2 (n=6). Cells were analyzed using a two-color antibody combination of CD3 and CD19 for (A) non-starved, (B) sham control and (C) Mox-treated mice.
